# Ramadan Fasting in Individuals with Metabolic Dysfunction-Associated Steatotic Liver Disease, Liver Transplant, and Bariatric Surgery: A Narrative Review

**DOI:** 10.3390/jcm13133893

**Published:** 2024-07-02

**Authors:** Musaab Ahmed, Mohamed H. Ahmed

**Affiliations:** 1 College of Medicine, Ajman University, Ajman P.O. Box 346, United Arab Emirates; 2 Department of Medicine and HIV Metabolic Clinic, Milton Keynes University Hospital NHS Foundation Trust, Eagelstone, Milton Keynes MK6 5LD, UK; mohamed.hassan-ahmed@mkuh.nhs.uk; 3 Department of Geriatric Medicine, Milton Keynes University Hospital NHS Foundation Trust, Eagelstone, Milton Keynes MK6 5LD, UK; 4 Honorary Senior Lecturer of the Faculty of Medicine and Health Sciences, University of Buckingham, Buckingham MK18 1EG, UK

**Keywords:** Ramadan fasting, non-alcoholic fatty liver disease, liver transplant, bariatric surgery

## Abstract

Metabolic dysfunction-associated steatotic liver disease is a growing worldwide pandemic. A limited number of studies have investigated the potential effect of Ramadan fasting on metabolic dysfunction-associated steatotic liver disease (MASLD). There is no single medication for the treatment of MASLD. There is a growing interest in dietary intervention as potential treatment for metabolic diseases including MASLD. The aim of this study was to discuss the epidemiology, pathogenesis, and risk factors of MASLD and the potential effects of Ramadan fasting on MASLD, liver transplant, and bariatric surgery. We searched PubMed and SCOPUS databases using different search terms. The literature search was based on research studies published in English from the year 2000 to the 2024. Thirty-two studies were included in this review. Ramadan fasting reduced body weight and improved lipid profile, anthropometric indices, fasting plasma glucose, plasma insulin, and inflammatory cytokines. Ramadan fasting improved risk factors of nonalcoholic fatty liver disease and might improve MASLD through weight reduction. However, further studies are needed to assess the safety and effectiveness of Ramadan fasting in liver transplant recipients and bariatric surgery.

## 1. Introduction

Metabolic dysfunction-associated steatotic liver disease (MASLD) is a range of liver conditions that includes simple steatosis and metabolic dysfunction-associated steatohepatitis (MASH). It can develop into cirrhosis and hepatocellular carcinoma. This condition is not caused by factors such as excessive alcohol intake and viral hepatitis, or other causes of fatty liver disease [1,2]. MASLD is considered the liver manifestation of metabolic syndrome [3]. MASLD development is shaped by a complex interaction between genetic and environmental factors. This disease is more common among males and obese and overweight individuals. MASLD is a growing worldwide pandemic. The worldwide prevalence of MASLD is about 25% [4]. Currently, there is no single medication for MASLD. However, resmetirom showed promising outcomes in the treatment of NASH and liver fibrosis [5]. A healthy lifestyle is crucial for both preventing and treating MASLD [2]. Multiple studies reported that dietary intervention might have a beneficial impact on liver and metabolic indicators in individuals with MASLD. These interventions include the Mediterranean diet [6], intermittent fasting, and Ramadan fasting [2]. Observing fasting throughout the holy month of Ramadan is obligatory for all physically fit Muslims who have reached puberty. The length of the daily fast during Ramadan varies across various countries and from year to year due to the difference between the Islamic and Gregorian calendars. It may range from a few hours to over twenty hours, starting from dawn and ending at twilight. During Ramadan, Muslims who are observing the fast are required to refrain from any kind of oral consumption, such as drinking, eating, or smoking, from before sunrise until after sunset. The majority of those who observe Ramadan take two meals each day: Suhoor, which is eaten before dawn to begin the fast, and Iftar, which is had around sunset to break the fast [7]. Ramadan fasting has many health benefits, including the reduction in body weight and composition and the improvement of the components of metabolic syndrome. Additionally, fasting during Ramadan improves blood glucose levels and decreases inflammatory markers and oxidative stress [2]. The aim of this study was to discuss the epidemiology, pathogenesis, risk factors of MASLD and the potential effects of Ramadan fasting on MASLD, liver transplant, and bariatric surgery.

## 2. Metabolic Dysfunction-Associated Steatotic Liver Disease

Metabolic dysfunction-associated steatotic liver disease (MASLD) is a rapidly spreading global epidemic. The epidemic of MASLD presents a serious public health issue [8]. The buildup of triglycerides and free fatty acids in the liver is largely caused by obesity and insulin resistance. Individuals diagnosed with non-alcoholic fatty liver disease (MASLD) have an increased susceptibility to death caused by cardiovascular and liver-related complications. Non-alcoholic fatty liver disease (MASLD) has emerged as a significant indication for liver transplantation [9].

MASLD is an encompassing term that includes several disorders, ranging from the basic buildup of fat in the liver to more advanced cases including steatosis accompanied by hepatitis, fibrosis, cirrhosis, and hepatocellular cancer, all without excessive alcohol use. MASLD consists of two main conditions: non-alcoholic fatty liver (NAFL) and metabolic dysfunction-associated steatohepatitis (MASH) [10]. Non-alcoholic fatty liver is defined as the accumulation of fat in the liver, affecting more than five percent of the liver tissue, without causing damage to the liver cells. Metabolic dysfunction-associated steatohepatitis (MASH) is a condition characterized by inflammation and cell death in the liver cells, occurring with the accumulation of fat (steatosis) [11]. The majority of patients diagnosed with metabolic dysfunction-associated steatotic liver disease (MASLD) exhibit obesity and insulin resistance, which significantly contributes to the development of metabolic syndrome [12,13]. MASLD is regarded as the liver manifestation of metabolic syndrome, a cluster of metabolic abnormalities such as central obesity, hypertension, hyperglycemia, hypertriglyceridemia, and low high-density lipoprotein. These abnormalities elevate the risk of cardiovascular disease, stroke, and type 2 diabetes [14].

## 3. Methods

**Figure 1:**
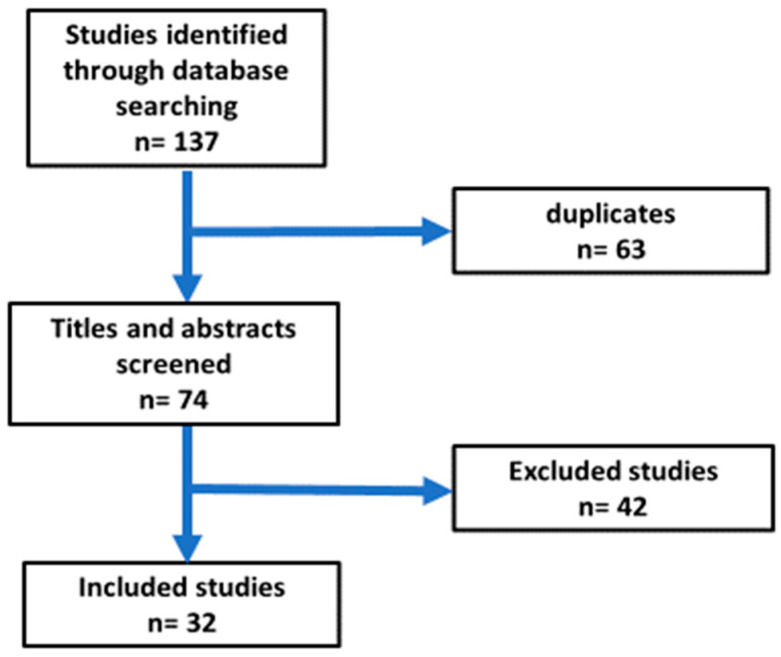
Flow chart for selection of articles included in the review.

This study was conducted as a narrative review. The authors searched SCOPUS and PubMed databases using the keywords: Nonalcoholic fatty liver, epidemiology, pathophysiology, and Ramadan fasting. The authors also conducted a search using a combination of the following terms: [Nonalcoholic fatty liver AND epidemiology AND pathophysiology] OR [Ramadan fasting AND Nonalcoholic fatty liver OR body weight OR obesity OR glucose OR diabetes OR lipid profile OR bariatric surgery OR liver transplantation AND clinical trials]. The literature search was based on research articles published in English from the 1 January 2000 to 30 January 2024; the abstracts and articles were then screened. Relevant articles were carefully reviewed, and data related to Ramadan fasting, study duration, the number of study participants, and effects on obesity, glycemia, dyslipidemia, bariatric surgery, and liver transplantation were extracted. Relevant references were included in the reference list. The total number of publications found in the initial search was 137. The total number of articles selected for the review was 32 (Figure 1). The following criteria were used for the selection of the articles.

### 3.1. Inclusion Criteria

Observational studies, randomized controlled trials, and experimental studies were included in the review. Publications written in English were eligible. All of the studies about nonalcoholic fatty liver or Ramadan fasting were included

### 3.2. Exclusion Criteria

Letters to the editor, commentaries, news, case reports, books, notes, theses, opinions, short surveys, repeated studies, conference abstracts, and publications written in a language other than English were removed.

All retrieved papers were imported into EndNote version 20 for the removal of duplicates. Then, two researchers fully screened abstracts and titles of the articles based on the eligibility criteria. The flow chart for the selection of the publications is shown in Figure 1.

## 4. Epidemiology of Metabolic Dysfunction-Associated Steatotic Liver Disease

Metabolic dysfunction-associated steatotic liver disease (MASLD) has emerged as the most prevalent liver disorder globally. Several researchers have attempted to determine the actual global prevalence of MASLD. However, a definitive and trustworthy prevalence rate is still unavailable because of discrepancies in the testing methods and study criteria used. The estimated prevalence rate in the Western world is between 20 and 30%, whereas in Asian nations it is considered to be around 5–18%. The incidence of metabolic dysfunction-associated steatotic liver disease (MASLD) is greater in men and increases with age. It is influenced by the diagnostic approach and the lifestyle choices of the population. In population studies, the diagnosis of metabolic dysfunction-associated steatotic liver disease (MASLD) is often conducted using ultrasonography, which tends to underestimate the prevalence of fatty liver. The Dallas Heart Study and the Dionysos Study revealed that 30% of individuals in the United States and 25% in Italy are affected by metabolic dysfunction-associated steatotic liver disease (MASLD). The incidence of Metabolic dysfunction-associated steatotic liver disease (MASLD) is 80–90% among people who are obese, up to 90% among patients with hyperlipidemia, and 30–50% among diabetic patients. The prevalence of this condition ranges from 3% to 10% among children, but it significantly increases to 40% to 70% in children who are obese. The global incidence of metabolic dysfunction-associated steatotic liver disease (MASLD) is rising owing to the consumption of high-fat diets and sedentary lifestyles. The primary risk factors for non-alcoholic fatty liver disease (MASLD) are being male, advancing age, obesity, insulin resistance, and the cardiometabolic abnormalities associated with metabolic syndrome [10,15].

## 5. Pathogenesis of Metabolic Dysfunction-Associated Steatotic Liver Disease

The precise pathogenic process of MASLD is complex and yet not fully understood. The primary characteristic of the condition is the buildup of free fatty acids and triglycerides, which is mostly caused by insulin resistance and obesity [16].

The pathophysiology of MASLD was explained using the two-hit model. The first hit encompasses the determinants of hepatic steatosis, which include a high-fat diet, sedentary lifestyle, hepatic lipid buildup, obesity, and insulin resistance [17]. The second hit involves the elements that trigger inflammation, cellular demise, and fibrosis, such as oxidative stress, endoplasmic reticulum stress, pro-inflammatory cytokines, and gut-derived bacterial endotoxin [18]. The two-hit model was deemed insufficient in comprehensively explaining the development of MASLD, since several variables work simultaneously in a genetically susceptible person [17]. This supports the proposition of a multi-hit model in 2010 [19]. Insulin resistance significantly contributes to the occurrence of oxidative stress, endoplasmic reticulum stress, proinflammatory cytokines, and gut-derived bacterial endotoxin. Hepatic steatosis leads to hepatic de novo lipogenesis, a reduction in adipose tissue lipolysis, and an increase in fatty acids in the liver [20]. Moreover, insulin resistance leads to changes in the synthesis and release of adipokines and inflammatory cytokines [21]. The buildup of triglycerides in the liver leads to an increase in the generation of reactive oxygen species, resulting in endoplasmic reticulum stress and mitochondrial dysfunction [22]. The gut microbiota is thought to have a crucial role in the development of metabolic dysfunction-associated steatotic liver disease (MASLD). The gut microbiota has an impact on the absorption and elimination of nutrients in the liver, as well as on hepatic inflammation. This is because they provide toll-like receptor ligands, which stimulate the liver to produce more proinflammatory cytokines. As a result, probiotics have been suggested as a potential treatment for MASH by altering the composition of intestinal bacteria [23]. Obesity, insulin resistance, diabetes mellitus, hypertension, hyperlipidemia, and hyperglycemia are potential complications that might arise in individuals after liver transplantation. The development of metabolic abnormalities may be attributed in part to the use of drugs such as corticosteroids, calcineurin inhibitors, and sirolimus after liver transplantation [24]. The post-liver transplantation features include the characteristics of metabolic syndrome, with MASLD serving as the hepatic expression of this condition. Therefore, it is typical for individuals to have a relapse or the development of new cases of metabolic dysfunction-associated steatotic liver disease (MASLD) or metabolic dysfunction-associated steatohepatitis (MASH) after a liver transplant [25]. Research has shown that 26.3% of individuals who have liver transplant surgery experience weight gain and become obese within a span of three years [26]. Additionally, a range of 10% to 64% of these transplant patients acquire diabetes mellitus. The precise mechanism underlying the development of diabetes mellitus in these patients remains unclear. However, it is well-established that the primary risk factors for diabetes include obesity, male gender, family history, hepatitis C virus infection, advanced age, and the use of high doses of immunosuppressive drugs [27]. Furthermore, the incidence of metabolic syndrome after liver transplantation is around 50–60% [28]. A cohort study including 170 transplant patients over a two-year period revealed that metabolic syndrome occurred in 33% of the patients [29]. The prevalence of MASLD after liver transplantation ranges from 18% to 40%. Polymorphisms in PNPLA3, which facilitate the breakdown of triglycerides, have been connected with an increased likelihood of developing metabolic dysfunction-associated steatotic liver disease (MASLD) following liver transplantation. These polymorphisms are also linked to pre-transplant obesity and MASLD. The occurrence of metabolic dysfunction-associated steatotic liver disease (MASLD) after liver transplantation may potentially contribute to the elevated cardiovascular mortality seen in these individuals [30].

## 6. Risk Factors of Nonalcoholic Fatty Liver Disease

Individuals with metabolic dysfunction-associated steatotic liver disease (MASLD) often exhibit features commonly seen in metabolic syndrome (MS), along with the corresponding risk factors for cardiovascular disease [31,32]. MASLD is strongly associated with metabolic syndrome and obesity. Type 2 diabetes mellitus (T2DM) and dyslipidemia are recognized as significant risk factors for MASLD [33]. Studies have demonstrated a greater incidence of cardiovascular disease (CVD) in individuals with metabolic dysfunction-associated steatotic liver disease (MASLD), both with and without diabetes [34,35]. Hence, MASLD is often linked to lifestyle, and there is evidence indicating that modifying lifestyle might lower transaminase levels and can therefore improve MASLD [36]. A study on patients with type 2 diabetes mellitus (T2DM) revealed that the prevalence of coronary, cerebrovascular, and peripheral vascular disease was significantly higher in subjects with MASLD [37]. Multiple studies have investigated the connection between MASLD and cardiovascular disease. These studies have consistently shown that cardiovascular disease poses a significant and immediate risk [38].

The association between MASLD and smoking is debatable [39]. A study conducted on rats with obesity revealed that exposure to cigarette smoke heightened the histological severity of metabolic dysfunction-associated steatotic liver disease (MASLD) [40]. A cross-sectional study conducted on individuals with non-alcoholic fatty liver disease (MASLD) reported that the prevalence of substantial liver fibrosis and advanced hepatic fibrosis was considerably greater among smokers compared to non-smokers [36]. Previous studies have identified smoking as an independent risk factor for the occurrence of metabolic dysfunction-associated steatotic liver disease (MASLD) [39].

## 7. Ramadan Fasting and Metabolic Dysfunction-Associated Steatotic Liver Disease

There is no specific medication for the treatment of MASLD. The first line in the treatment of patients with MASLD is lifestyle modification and weight reduction. These patients should be advised to perform regular exercise and to consume a healthy diet [41]. Several studies indicated that adhering to a Mediterranean diet, which involves consuming less carbs (particularly sweets and refined carbohydrates) and increasing the consumption of monounsaturated and omega-3 fatty acids, might effectively decrease liver fat. If lifestyle adjustments fail to produce the expected outcomes, the use of supplementation with vitamin E therapy should be considered as a therapeutic option [42]. Although the influence of lifestyle adjustments on MASLD is undeniable, it is exceedingly challenging to attain long-term weight management outcomes through dietary modification and physical exercise alone [43]. Administering Glucagon-like peptide-1 (GLP-1) receptor agonists has shown significant improvement in hepatic outcomes among patients diagnosed with metabolic dysfunction-associated steatotic liver disease (MASLD) [44,45]. Resmetirom, a thyroid hormone receptor agonist, is administered at daily doses of either 80 mg or 100 mg. Resmetirom was used in a 36-week phase 2 trial in patients with MASH and fibrosis and produced a reduction in liver fat content and improvement in liver function tests and lipid metabolism parameters. In addition, the lipid profile and fibrosis markers improved without influencing body weight [46,47]. Resmetirom was administered to patients with a non-invasive diagnosis of MASLD at a dosage of 100 mg per day for 52 weeks in a phase 3 trial. A non-invasive test confirmed that liver fibrosis improved in approximately 20% of treated patients and 10% of placebo-treated patients, while liver lipid content improved in approximately 50% of treated patients and 8% of placebo-treated patients. Resmetirom also improves liver enzymes, lipid metabolism parameters, and inflammatory biomarkers in the absence of significant safety concerns. The primary adverse events are diarrhea and nausea [47]. Resmetirom use was associated with MASH resolution and an improvement in liver fibrosis by at least one stage in a phase 3 study in MASH patients with fibrosis (MAESTRO-NASH, NCT03900429). In this study, 966 patients with biopsy-confirmed MASH and a fibrosis stage of F1B, F2, or F3 were randomly assigned in a 1:1:1 ratio to receive once-daily resmetirom (322 in the 80 mg resmetirom group, 323 in the 100 mg resmetirom group, and 321 in the placebo group) over a 52-week period. In comparison to the placebo group, 25.9% of patients in the 80 mg resmetirom group and 29.9% of patients in the 100 mg resmetirom group achieved MASH resolution without fibrosis worsening. The NAFLD activity score did not worsen in 24.2% of the patients in the 80 mg resmetirom group and 25.9% of those in the 100 mg resmetirom group. Fibrosis improved by at least one stage. LDL cholesterol levels decreased by −13.6% and −16.3% in the 80 mg and 100 mg resmetirom groups, respectively, at week 24, compared to 0.1% in the placebo group. Resmetirom was associated with a higher incidence of diarrhea and nausea than the placebo. The three groups had a comparable incidence of significant adverse events, which was no more than 13% [48]. Resmetirom was granted accelerated approval by the Food and Drug Administration on 14 March 2024, as the first-ever drug to be approved for the treatment of MASH with stage F2 or F3 fibrosis, as a result of these findings [46]. Resmetirom treatment resulted in increased costs of US$66,764 per patient, while increasing quality-adjusted life-years by 1.24 [49].

Pemafibrate, a PPARα agonist, has been evaluated in patients with MASH who have been screened using MRI and ALT elevation. Even though the percentage change in liver fat content by MRI at week 24 was only −5.3% compared to −4.2% in controls, liver stiffness by MRI substantially decreased at week 48 and was maintained at week 72 (treatment difference −6.2%) [50].

The engineered analog of recombinant human FGF19, Aldafermin (NGM282) is a 190-amino-acid peptide with a homology of 95.4%. Aldafermin has the potential to modulate metabolic homeostasis and block the synthesis of BAs. Aldafermin lowered hepatic lipid accumulation by 7.7% in a 24-week phase 2b trial; however, liver fibrosis did not ameliorate in patients with MASH-related stage 2 or 3 fibrosis [51]. Aldafermin was well tolerated in an additional phase 2b trial, and there was no significant dose-dependent response in fibrosis [52].

A recent clinical trial was conducted to investigate the effects of Policaptil Gel Retard (PGR) on patients with type 2 diabetes mellitus and metabolic syndrome. The study involved 245 participants who were randomly assigned to either receive PGR or a placebo for a duration of 24 weeks. During the time of the study, participants were permitted to follow a low-calorie diet and engage in enhanced physical activity. Introducing PGR with lifestyle modifications enhanced lipid and glucose metabolism-related factors, such as insulin resistance, and notably decreased both visceral fat and liver fat content, as well as the severity of liver fibrosis. The impact of PGR was probably associated with a decrease in the maximum levels of blood glucose and insulin after a meal [53].

Another option that can be used for the treatment of patients with MASLD is bariatric surgery. The advantages of this intervention in terms of weight reduction and the improvement of several metabolic illnesses, such as type 2 diabetes mellitus (T2DM), have been well documented. Furthermore, it has resulted in a much-improved long-term survival rate [54,55]. Metabolic dysfunction-associated steatotic liver disease has become an indication for liver transplantation [9].

Few studies evaluated the effects of Ramadan fasting on nonalcoholic fatty liver. An experimental study by Alasmari et al. conducted on 48 rats showed that Ramadan fasting caused a significant reduction in body weight, plasma levels of cholesterol, LDL, and triglycerides, and the enzymes aspartate transaminase and alanine transaminase [8]. A study by Ebrahimi et al. included 83 patients with MASLD and reported that Ramadan fasting caused a significant reduction in total cholesterol and a reduction in the Visceral Adiposity Index (VAI) and the Atherogenic Index of Plasma (AIP) in the patients. The authors concluded that Ramadan fasting might be useful in the management of patients with MASLD [56]. Mari et al.’s study on 155 patients with MASH showed that Ramadan fasting improved body weight, insulin sensitivity inflammatory markers, and noninvasive measures for MASH severity assessment [57]. An observational study by Aliasghari et al. included 83 patients and demonstrated that Ramadan fasting improved anthropometric indices, fasting plasma glucose, plasma insulin, and inflammatory cytokines in patients with MASLD [58]. Ramadan fasting might improve MASLD through weight reduction. It has been reported that weight reduction causes an improvement in all of the segments of histological activity in patients with nonalcoholic steatohepatitis [59]. Badran et al. examined the impact of Ramadan fasting on 98 individuals diagnosed with metabolic dysfunction-associated steatotic liver disease (MASLD). The authors found that Ramadan fasting caused significant enhancements in the biochemical, anthropometric, and ultrasound parameters of individuals with metabolic dysfunction-associated steatotic liver disease (MASLD), particularly during the first stages and among those at risk of developing diabetes [60]. Mari et al. investigated the impact of Ramadan fasting on individuals with MASH. They showed that fasting had the potential to enhance the severity of MASH, insulin sensitivity, and inflammation [57]. Ramadan fasting might be an effective dietary intervention for MASLD [2]. Further clinical studies are needed to confirm the effect of Ramadan fasting on MASLD.

## 8. Ramadan Fasting and Risk Factors of Metabolic Dysfunction-Associated Steatotic Liver Disease

### 8.1. Ramadan Fasting and Obesity

Eleven studies demonstrated the effects of Ramadan fasting on body weight and body fat. A study conducted by Rahman et al. revealed that fasting during Ramadan can significantly decrease body weight and increase HDL among adult males who are in good health [61]. A study conducted by Nommsen-Rivers provided evidence that Ramadan intermittent fasting can lead to a reduction in visceral fat thickness in pregnant women, while not having any adverse effects on fetal development or amniotic fluid levels. However, this evidence is inadequate to establish the safety of intermittent fasting for pregnant women. Moreover, the study has suggested that maternal obesity may result in postponed lactation [62]. A study conducted by Madkour et al. demonstrated that observing fasting during Ramadan can improve oxidative stress and unfavorable metabolic disorders among obese patients who do not have diabetes [63]. Sadiya et al. reported that Ramadan fasting can reduce waist circumference and body weight and in patients with MetS [64]. Zouhal et al. showed that Ramadan intermittent fasting improved body composition index in healthy obese males and increased plasma levels of leptin and decreased plasma levels of GLP-1, PYY, and CCK. The authors concluded that intermittent fasting can combat obesity [65]. A recent study conducted by Madkour et al. on 57 overweight and obese individuals showed that Ramadan fasting produced a significant reduction in body weight, fat mass, waist and hip circumference, LDL, and triglycerides and an increase in HDL-C. It also caused reductions in both IL-6 and TNF-alpha and an increase in plasma IL-10 levels [66]. A study by Abdullah et al. involving 21 healthy Muslims showed that Ramadan fasting produced a significant reduction in body mass index, body weight, waist circumference, insulin resistance, visceral and subcutaneous fat, and fat thickness [67]. A study conducted by Maaloul et al. involving 20 obese individuals demonstrated that Ramadan fasting with concurrent training produced a greater improvement in body weight, fat percentage, and waist circumference, a greater decrease in triglycerides, total cholesterol, and LDL, and a reduction in the serum levels of CRP [68]. A study by Celik et al., 2022, involving 32 healthy individuals in Turkey, showed that Ramadan fasting produced a significant decrease in body mass index, plasma levels of C-reactive protein (CRP), and tumor necrosis factor-alpha. Fasting blood glucose levels increased with no effect reported on lipids [69]. A study by Ouselati et al., 2022, including 52 individuals with type 2 diabetes mellitus, demonstrated that Ramadan fasting produced a significant decrease in weight, waist circumference, body mass index, fat body mass, insulin levels, and CRP levels. There were no significant changes in fasting blood glucose and blood pressure [70]. A study by Farag et al., 2020 in Iraq, including 120 hypertensive patients, showed that Ramadan fasting produced a significant reduction in blood pressure, body mass index, body weight, waist circumference, total cholesterol, and LDL. There was no significant change in LDL, while triglycerides decreased significantly following Ramadan fasting [71]. Ramadan fasting caused a reduction in body fat and body weight.

### 8.2. Ramadan Fasting and Type 2 Diabetes

Seven studies reported the effects of Ramadan fasting on glycemia. A recent study by Tahapary et al. showed that Ramadan fasting caused a significant reduction in ICAM-1 levels, a marker of endothelial function, in both type 2 diabetes mellitus and non-DM patients [72]. A study by Abdullah et al. involving 21 healthy Muslims showed that Ramadan intermittent fasting produced a significant reduction in insulin resistance and subcutaneous and visceral fat thickness [67]. A study by Ouselati et al., 2022, including 52 individuals with type 2 diabetes mellitus, demonstrated that Ramadan fasting produced a significant decrease in weight, body mass index, waist circumference, fat body mass, insulin levels, and CRP levels. There was no significant change in fasting plasma glucose and blood pressure. The authors concluded that Ramadan fasting may have favorable effects on inflammation and metabolic profile and might worsen the glycemic control in individuals with types 2 diabetes [70]. Mohammadzadeh et al., 2022, in a study including 30 healthy individuals, showed that Ramadan fasting significantly reduced serum levels of HDL, LDL, LDL/HDL ratio, and total cholesterol. There was no significant decrease in triglycerides and fasting blood glucose levels [73]. Ben Ahmed et al. conducted a study in Tunisia involving 84 patients to evaluate the impact of Ramadan fasting on patients with stable coronary artery disease and showed that Ramadan fasting produced an improvement in the plasma concentrations of triglycerides, cholesterol, low-density lipoprotein cholesterol, and apoprotein and a significant reduction in fasting blood glucose, insulin level, insulin resistance index, and plasma CRP level [74]. Similarly, Bener et al.’s study including 1246 diabetic patients showed that Ramadan fasting produced a significant improvement in blood glucose and HbA1c, a significant reduction in total cholesterol and triglycerides, a significant increase in HDL-C (in females), and a significant reduction in LDL-C (in males) and systolic and diastolic blood pressure [75]. Prasetya et al. showed that Ramadan fasting significantly improved insulin sensitivity and reduced leptin levels, and did not produce significant change in blood glucose level in healthy men [76]. The available evidence exhibits inconsistent support for an improvement in fasting plasma glucose levels, possibly attributable to a latency period or overlooked glucose level fluctuations throughout the day [77,78]. The observed variations in the results of these investigations could potentially be adequately explained by the differing cohorts under examination, namely, those who are classified as obese versus those who are not, thereby implying a discernible effect based on subgroup distinctions.

### 8.3. Ramadan Fasting and Dyslipidemia

Seven studies reported the effects of Ramadan fasting on lipid profile. The observance of Ramadan fasting was associated with favorable lipid profiles, as evidenced by improved HDL, LDL, triglyceride, and VLDL levels. This was further reflected in a reduction in the average Framingham risk score from 13.8 to 10.8 [79]. Madkour et al. showed that Ramadan fasting produced a significant reduction in body weight, fat mass, waist and hip circumference, LDL, and triglycerides and an increase in HDL-C [66]. Similarly, the Bener et al. study including 1246 diabetic patients showed that Ramadan fasting produced a significant reduction in total cholesterol and triglycerides, a significant increase in HDL-C (in females), and a significant reduction in LDL-C (in males) [75]. A study conducted by Maaloul et al. involving 20 obese individuals demonstrated that Ramadan fasting with concurrent training produced a greater decrease in triglycerides, total cholesterol and LDL and a reduction in the serum levels in CRP [68]. Mohammadzadeh et al., 2022, in a study including 30 healthy individuals, showed that Ramadan fasting significantly reduced serum levels of LDL, HDL, LDL/HDL ratio, and total cholesterol. There was no significant decrease in triglycerides and fasting blood glucose levels [73]. The Ben Ahmed et al. study in Tunisia involving 84 patients to evaluate the impact of Ramadan fasting on patients with stable coronary artery disease showed that Ramadan fasting produced an improvement in the plasma concentrations of triglycerides, cholesterol, low-density lipoprotein-cholesterol, and apoprotein A [74]. The Farag et al., 2020 study in Iraq including 120 hypertensive patients showed that Ramadan fasting produced a significant reduction in blood pressure, body weight, body mass index, and waist circumference. Total cholesterol and LDL did not change significantly while triglycerides decreased significantly following Ramadan fasting [71]. It has been proposed that intermittent fasting improves lipid profiles through the nuclear expression of peroxisome proliferator-activated receptor gamma coactivator 1alpha and peroxisome proliferator-activated receptors in the liver, leading to increased fatty acid oxidation, ApoA production, decreased ApoB synthesis, decreased hepatic triglycerides, and the production of VLDL. All of these effects would cause a reduction in serum levels of LDL-C, VLDL, and small dense LDL-C [80].

### 8.4. Ramadan Fasting and Bariatric Surgery

There is a scarcity of published data on the impact of Ramadan fasting on bariatric surgery [81] (Table 1).

### 8.5. Ramadan Fasting and Liver Transplantation

Only two published studies addressed the topic of Ramadan fasting and liver transplantation (LT); one was conducted in Qatar and the other one was conducted in Egypt. Montasser et al., 2020 conducted a prospective, non-controlled, observational study involving 45 individuals who wanted to observe the Ramadan fasting at the Ain Shams Center for Organ Transplantation in Cairo, Egypt. A total of 37 patients (82.2%) from the study group, who were advised to consume more than 3 L of fluids per day and followed a modified immunosuppressant regimen, successfully observed the fasting period throughout Ramadan. While three patients (6.6%) had interrupted fasting due to unrelated factors, five patients (11.1%) had to discontinue fasting due to an unsatisfactory increase in their renal functions. The laboratory and clinical status related to the liver were not modified by fasting. The observed alterations in these parameters were temporary and not significant. A substantial statistical difference was seen in the blood creatinine level between the pre- and post-fasting phases (*p* = 0.004). The authors concluded that liver transplant recipients who adhere to a modified immunosuppressive strategy, maintain adequate hydration intake, and undergo frequent follow-up may safely practice Ramadan fasting [86]. In 2018, Derbala et al. conducted a retrospective study of 96 patients who had liver transplants at Hamad Hospital in Qatar. No statistically significant disparity in tacrolimus level (*p* = 0.96) was observed between those who practiced Ramadan fasting and those who did not fast for Ramadan. Notably, there were elevated levels of albumin, total proteins, cholesterol, creatinine, hemoglobin, and platelet count in both before and after Ramadan, as opposed to during Ramadan. These findings suggest temporary fluctuations. No significant disparities were seen in any of the biochemical and hematological indices between individuals who underwent fasting and those who did not. It was determined that individuals who have steady graft function and do not have cirrhosis may safely undergo Ramadan fasting [87]. Further research studies with larger sample sizes are needed to assess the safety of Ramadan fasting in individuals with a liver transplant. Until such studies become available, it is extremely prudent to advise patients with liver transplant not to fast Ramadan.

## 9. Conclusions

Ramadan fasting improves risk factors of metabolic dysfunction-associated steatotic liver disease. Ramadan fasting may have the potential to improve metabolic parameters of metabolic dysfunction-associated steatotic liver disease (Table 2). Further studies are needed to confirm the safety and effectiveness of Ramadan fasting on patients with metabolic dysfunction-associated steatotic liver disease, liver transplant, and bariatric surgery.

## Figures and Tables

**Table 1:** Summary of published studies on the impact of Ramadan fasting on bariatric surgery.

Authors	Main Finding	Reference
Kermansaravi et al., 2021	Delphi consensus study recommended the following: Specific nutritional assistance should be given during fasting and it is recommended not to fast in the case of intolerance. It is necessary for the patient to consult with both the surgeon and dietitian prior to fasting. The initiation of fasting might be delayed for a minimum of 6–12 months after the surgery. Proton pump inhibitors are advised if fasting is planned in less than 6 months after the surgery.	[82]
Al-Ozairi et al., 2015	Fasting during Ramadan after bariatric surgery is well tolerated. It is crucial to educate patients about the need for adherence to medication and consuming enough protein. No episode of hypoglycemia in the case of excellent adherence to medication and no change in fluid or protein intake. A total of 52.7% of patients experienced a slight weight loss after fasting during Ramadan, while approximately 18% gained weight, and more than 22% did not experience any change in weight after Ramadan.	[83]
Tat et al., 2020	The authors concluded that there is no significant adverse risk associated with undergoing bariatric surgery before, during or after the period of Ramadan (the total number of patients was 542).	[84]
Sherf Dagan et al., 2017, Kermansaravi et al., 2021	It is advisable to postpone fasting for a period of six to eighteen months after bariatric surgery.	[82,85]

**Table 2:** List of potential benefits of fasting in patients with MASLD, liver transplant, and bariatric surgery.

Potential Significant Benefits of Ramadan Fasting		
MASLD	Liver Transplant	Bariatric Surgery
Reduction in body weight. Decreased plasma cholesterol, LDL, and triglycerides. Decreased liver enzymes: aspartate transaminase and alanine transaminase. Decreased abdominal obesity. Improved insulin sensitivity. Improved inflammatory markers and noninvasive measures for NASH severity assessment.	Further studies are needed to assess the effectiveness and safety of Ramadan fasting in patients with liver transplant. So far, two studies were conducted with small sample sizes. Further studies are needed with a larger sample size.	No significant weight change expected during Ramadan. No significant danger associated with undergoing bariatric surgery before, after, and during the period of Ramadan.
